# Supplementary material for: Synthetic GPR84 Agonists in Colorectal Cancer: Effective in THP-1 Cells but Ineffective in BMDMs and MC38 Mouse Tumor Models
Source: Int J Mol Sci. 2025 Jan 9;26(2):490. doi: 10.3390/ijms26020490 (PMC11764671; doi:10.3390/ijms26020490)
Supplement: Supplementary file 1 [file ijms-26-00490-s001.zip › ijms-3390351-supplementary.pdf]

International Journal of
Molecular Sciences

Supplementary Material and Methods

1. *Production and testing of in-house MCSF*

In-house produced MCSF was obtained from the supernatant of the murine fibroblast cell line L929. Therefore, L929 cells were expanded in 300 cm2 cell culture flasks (TPP) at 37°C, 5% CO_2 in 50 mL Dulbecco's Modified Eagle Medium (DMEM; 1000 mg/l D-glucose, + L-Glutamine, + Sodium Pyruvate) (Thermo Fisher Scientific) supplemented with 10% FCS and 1 mM L-Glutamine. Supernatant was harvested as soon as the cells reached 100% confluency, sterile filtered, aliquoted and the optimal concentration was evaluated in a differentiation test. Therefore, bone marrow cells were differentiated as described in the main methods section but with different concentrations of L929 supernatant: 5%, 10%, 15% and 20%. Differentiation of bone marrow derived cells was documented using a Nikon Eclipse Ts2 microscope. After 7 days, cells were detached, collected in FACS tubes, counted, and expression of the pan-macrophages marker F4/80 among the different conditions was analyzed using flow cytometry. For this aim, the cells were re-suspended in PBS (0.1 mL, pH 7.4), and stained with the Zombie NIRTM Fixable Viability Kit (#423106, BioLegend) diluted 1:500 and an anti-F4/80 AlexaFluor647 conjugated antibody (clone CI:A3-1, #MCA497A647, BioRad) diluted 1:200 in PBS for 20 min at 4° in the dark. Cells were washed with PBS (0.5 mL, pH 7.4) and centrifuged at 1700 rpm for 5 min. The supernatant was removed, and cells were fixed in 0.1% PFA/PBS (0.2 mL, pH 7.4). The samples were measured using a BD LSRFortessa™ Cell Analyzer (BD Biosciences) cytometer equipped with 405 nm, 488 nm, 561 nm, and 640 nm laser lines (BD Bioscience) and the FACSDiva Software (BD Bioscience). Data was analyzed by manual gating using FlowJo v10.10 (BD Bioscience).

1. *Specificity test of the anti-GRP84 antibody*

The specify of the primary antibody was tested by mixing murine T-cells with BMDMs 1:1. CD3+ T-cells were isolated from spleen using the EasySepTM mouse CD3+ isolation kit (Stemcell Technologies). The cell mixture was washed twice with PBS (0.5 mL, pH 7.4) and incubated with anti-GPR84 antibody (rabbit pAb, #DF2769-200UL, Affinity Bioscience) diluted 1:800 in PBS (0.1 mL, pH 7.4) for 1 h at 4°C. Cells incubated with secondary but no primary antibody was used as secondary antibody control (Ctrl). Cells were centrifuged at 1700 rpm for 5 min, washed twice with PBS (0.5 mL, pH 7.4) and incubated with an anti-rabbit AlexaFluor594 conjugated secondary antibody (goat pAb, #A11081, Thermo Fisher Scientific) 1:1000, Zombie NIRTM Fixable Viability Kit diluted 1:500 (#423106, BioLegend), CD3-BV785 (clone 145-2C11, #100355, BioLegend) diluted 1:150 and F4/80-AlexaFluor647 (clone CI:A3-1, #MCA497A647, BioRad, Hercules, United States) diluted 1:200 in PBS (0.1 mL, pH 7.4) for 1 h at 4°C. Cells incubated with no primary anti-GPR84 were used as secondary antibody control. Cells were centrifuged at 1700 rpm for 5 min, washed twice with PBS (0.5 mL, pH 7.4) and fixed in 0.1% PFA/PBS (0.222 mL, pH 7.4). The samples were measured using a BD LSRFortessa™ Cell Analyzer (BD Biosciences) cytometer equipped with 405 nm, 488 nm, 561 nm, and 640 nm laser lines (BD Bioscience) and the FACSDiva Software (BD Bioscience). Data was analyzed by manual gating using FlowJo v10.10 (BD Bioscience).

2. *Synthesis and quality control of GRP84 agonists*

6-OAU and ZQ-16 were synthetized by CISTIM, Leuven, Belgium. Chemistry: All reagents and solvents were purchased from commercial sources and used without further purification. TLC was carried out with Macherey-Nagel Alugram Sil G/UV254 plates. TLC plates were revealed with UV light. 1H NMR spectra were recorded on a 500 MHz Bruker spectrometer. Proton chemical shifts are reported in parts per million (δ) using TMS as a standard. The purity of all compounds screened in biological assays was >95% by HPLC. Purity was determined by LC−MS recorded on a system consisting of a Waters Acquity UPLC system (detection: diode array detector [DAD] and TQ detector), using a Waters BEH C18 (1.7 μm, 2.1×50 mm) column and (10 mM CH3COONH4 in 95% H_2O + 5% CH3CN) / CH3CN eluent.

Synthesis of 2-(hexylthio)-6-hydroxypyrimidin-4(3H)-one (ZQ-16): To a stirred solution of sodium hydroxide (3.469 g, 86.716 mmol, 2.5 eq.) and 2-thioxodihydropyrimidine-4,6(1H,5H)-dione (5.0 g, 34.687 mmol, 1.0 eq.) in water (20 mL) was treated with 1-bromohexane (5.823 mL, 41.624 mmol, 1.2 eq.) at room temperature. The resulting reaction mixture was stirred at 70oC for 8 h. The progress of the reaction mixture was monitored by TLC (mobile phase: 5% methanol: DCM, UV active). After completion of the reaction, the reaction mass was diluted with water (10 mL). The residue was acidified with aqueous hydrochloric acid (1N) until reaching a pH 0.5-1.5 and the contents were stirred for 60-90 min. The solid was filtered using a Buchner funnel and the solid was washed with water (20 mL) and petroleum ether (10 mL). The mixture was dried under vacuum to obtain 2-(hexylthio)-6-hydroxypyrimidin-4(3H)-one (1.3 g), which was

triturated with H_2O (at 50°C), and then triturated with 50% EtOAc/hexane (yield: 1.2 g with LCMS 98%). Recrystallization was attempted with different solvents (methanol, ethanol, ACN) but the compound was not crystallizing. Then the compound was taken up in 5% methanol: DCM, stirred for 1 h at room temperature to afford 2-(hexylthio)-6-hydroxy-pyrimidin-4(3H)-one (1.04 g, ZQ-16). 1H-NMR: (500 MHz, DMSO-d6) δ ppm 12.20 (s, 1 H), 11.35 (s, 1 H), 5.10 (s, 1 H), 3.08 (t, 2 H), 1.61 (t, 2 H), 1.37-1.24 (m, 6 H), 0.86 (t, 3 H); UPLC-MS (Reaction Neut 20% ESI_1): Rt = 2.48 min (100.0%); m/z 229.1 [M+H]+, m/z 227.2 [M-H]-.

Synthesis of 6-(octylamino)pyrimidine-2,4(1H,3H)-dione (6-OAU): A suspension of 6-chlorouracil (200 mg, 1.35 mmol) and n-octylamine (891 mg, 6.76 mmol) was refluxed in 1-butanol (3.0 mL) at 125oC for 18 h. The progress of the reaction mixture was monitored by TLC (mobile phase: 5% methanol: DCM, UV active). After completion, the reaction was cooled to room temperature, and half of the solvent was removed by evaporation under reduced pressure. The precipitated solid was filtered off, washed with 1-butanol (~20 mL), followed by diethyl ether (~20 mL), and the final compound recrystallized from ethanol, and finally dried under vacuum at room temperature to afford 6-(octylamino)-1H-pyrimidine-2,4-dione (170.0 mg, 6-OAU). 1H-NMR: (500 MHz, DMSO-d6) δ 10.14 (s, 1H), 9.80 (s, 1H), 6.07 (s, 1H), 4.39 (s, 1H), 3.01 – 2.96 (m, 2H), 1.49 – 1.46 (m, 2H), 1.35 – 1.23 (m, 11H), 0.88 – 0.85 (m, 3H); UPLC-MS (Reaction Neut 20% ESI_1): Rt = 2.52 min (99.0%); m/z 240.2 [M+H]+, m/z 238.0 [M-H]-.

3. *Confirmation of CD47 expression on MC38 cells*

MC38 cells were collected in FACS tubes, centrifuged at 1700 rpm for 5 min and washed twice with PBS (0.5 mL, pH 7.4). Thereafter, cells were re-suspended in PBS (0.1 mL, pH 7.4), and incubated with 10 µg/mL anti-CD47 antibody (#16-0471-81, Thermo Fisher Scientific) for 1 h at 37°C, 10% CO_2. The isotype control condition was incubated with Rat IgG2a kappa Isotype Control (eBR2a) (eBiosciene) instead. Cells were centrifuged at 1700 rpm for 5 min, washed twice with PBS (0.5 mL, pH 7.4), re-suspended in PBS (0.1 mL, pH 7.4) and incubated with anti-rat antibody AlexaFluor594 conjugated secondary antibody (goat pAb, #A11007, Thermo Fisher Scientific, diluted 1:1000) and with the Zombie NIRTM Fixable Viability Kit (#423106, BioLegend, diluted 1:500) in PBS for 1 h. Cells were centrifuged at 1700 rpm for 5 min, washed twice with PBS (0.5 mL, pH 7.4) and fixed in 0.1% PFA/PBS (0.2 mL, pH 7.4). The samples were measured using a BD LSRFortessa™ Cell Analyzer (BD Biosciences) cytometer equipped with 405 nm, 488 nm, 561 nm, and 640 nm laser lines (BD Bioscience) and the FACSDiva Software (BD Bioscience). Data was analyzed by manual gating using FlowJo v10.10 (BD Bioscience).

4. *ALPHAScreen cAMP assay – human GPR84*

cAMP Hunter™ CHO-K1 GPR84 Gi Cell Line (DiscoverX) was used to determine compound potency with human GPR84. Intracellular cAMP levels were measured using the ALPHAScreen cAMP assay (Perkin Elmer).

5. *In-vitro plasma protein binding (PPB) assay*

In vitro evaluation of the compounds for protein binding in Human (hPPB)/Mouse plasma (mPPB) was performed using the rapid equilibrium dialysis method, by Aragen Life Sciences, Pvt. Dt. Benaluru, India. 10 mM stocks of the test compounds (6-OAU/ZQ-16) were prepared in DMSO. From the 10mM stocks, 100 µM dilutons were prepared in methanol. 1 µM working stocks were prepared in plasma by spiking 5 µL of 100 µM stock into 495 µL of plasma. An aliquot of 200 µL of plasma containing test compound was added to the donor well and 350 µL of PBS was added to the receiver well. The plate was incubated at 37 0C in thermomixer at 400 rpm for 5 hours. The samples were matrix equilibrated with opposite matrix (25 µL of plasma/buffer sample was matched with 25 µL of blank buffer/plasma). Matrix matched samples were precipitated with 300 µL of acetonitrile containing internal standard. Samples were vortexed at 850 rpm for 5 min and centrifuged at 4000 rpm for 10 min. Supernatant was separated, diluted 2-fold with water and analyzed in LC-MS/MS. Control samples were processed immediately after the preparation of plasma working stock solutions. These samples served as a measure for calculating the percentage recovery of test compounds. The percentage of bound/unbound fraction was calculated by the following equations: % Unbound = 100 *Receiver area ratio / Donor area ratio; % Recovery = 100 * (Receiver area ratio + Donor area ratio) / Average Area ratio at 0 min *100.

6. *In-vitro metabolic stability assays*

In vitro evaluation of the compounds for metabolic stability was performed using Human (HLM)/Mouse liver microsomes (MLM), by Aragen Life Sciences, Pvt. Dt. Benaluru, India. 10 mM stocks of the test compounds (6-OAU/ZQ-16) were prepared in DMSO and diluted with water: acetonitrile (1:1) to a concentration of 1 mM. 100 µM working solutions were prepared by further dilution with water: acetonitrile (1: 1). Pre-incubation mixture: A pre-incubation mixture was prepared by combining 2.5 µL of the test compound, 75 µL of liver microsomes at a concentration of 1.666

mg/mL, and 85 μL of 100 mM potassium phosphate buffer. The mixture was pre-incubated for 10 minutes at 37 0C. Subsequently, the pre-incubation mixture was incubated for another 120 min by using 32.5 μL of the pre-incubation mixture and 17.5 μL of 100 mM potassium phosphate buffer at 37 0C without the co-factor (NADPH). For the zero-minute sample, 16.25 μL of the pre-incubation mixture was mixed with 200 μL of acetonitrile containing the internal standard, and 8.75 μL of the cofactor. Incubation mixture: The remaining pre-incubation mixture was mixed with 62 μL of the co-factor (NADPH 2.85 mM) and incubated for 120 min at 370C. Sample preparation: Samples were prepared by mixing 25 μL of the incubation mixture with 200 μL of acetonitrile that contained an internal standard. The mixture was vortexed for 5 min at 1200 rpm and centrifuged for 10 min at 4000 rpm. Supernatant was diluted 2 fold with water and injected on LC-MS/MS. Data analysis: % remaining of the test substance = (Peak Area ratio at time (min)) *100/(Peak Area Ratio at 0 min); Half-life: T1/2 (min) = 0.693/ Kel; CLint protein (μL/min/mg protein) =ABS (Kel/Protein Concentration)*1000; CLint invivo (mL/min/kg body wt.) =(CLint protein*microsomal protein yield*Liver factor)/1000; CLinvivo well stirred model (mL/min/kg body wt.) = [(CLint invivo*QH)/(CLint invivo+QH)]; %QH = ((CLinvivo well stirred model*100)/(QH))

7. *Selectivity - rodent GPR84*

Selectivity tests were performed at EuroscreenFast, Gosselies, Belgium. ZQ-16 was tested for agonist activity at the human FFA1 (GPR40) (FAST-0110A), FFA2 (GPR43) (FAST-0112A), FFA3 (GPR41) (FAST-0111A), FFA4 (GPR120) (FAST-0113A) and HCA3 (FAST-0351A) receptors at 10 μM, in duplicate. ZQ-16 was also tested for agonist activity at the mouse GPR84 receptor (FAST-993C) in a cAMP assay at 1, 3, 10, 30, 100, 300, 1000, 3000, 10000, and 30000 nanomolar concentrations, in duplicate. The compound was tested using the genetically engineered cell lines shown in Supplementary Table 1. Results are indicated in Supplementary Table 2.

8. *Safety scan*

Safety tests were performed by Eurofins DoscoverX corporation, San Diego, California, United States of America. A SAFETYscan E/IC50 ELECT test was performed. Data is shown in Supplementary Table 3.

9. *Pharmacokinetic analysis*

Pharmacokinetic (PK) studies including 6-OAU and ZQ-16 were performed by Aragen Life Sciences Pvt. Ldt., Benaluru, India. Female C57Bl/6 mice with a weight range between 20-35 g were used. For the 6-OAU PK study n=3 mice per group were used. For the ZQ-16 PK study n=9 mice per group were used. The compounds were injected either intravenously (IV) or intraperitoneally (IP) or applied via oral gavage (PO). Vehicles, administration doses and time points for blood sampling are shown in Supplementary Tables 4 and 5 for each compound. Plasma concentration (ng/mL) of the respective compound was determined using a Phoenix WinNonlin® (build 8.1.0.3530) according to the standards and protocol of the CRO. Data is shown in Supplementary Tables 6 and 7.

Supplementary Tables

Supplementary Table S1. cAMP activity test

Agonist	cAMP Activity (nM)
6-OAU	60.6
ZQ-16	19.3

Supplementary Table S2. Protein plasma biding and metabolic stability

**PPB 6-OAU**	hPPB (%B)/%recovery	100/24.91
	mPPB (%B)/%recovery	100/15.91
**Metabolic stability 6-OAU**	Cl_{int} HLM (μL/min/mg prot)	1664.1
	Cl_{int} MLM (μL/min/mg prot)	52.3
**PPB ZQ-16**	hPPB (%B)/%recovery	100/0.71
	mPPB (%B)/%recovery	unstable
**Metabolic stability ZQ-16**	Cl_{int} HLM (μL/min/mg prot)	3.2
	Cl_{int} MLM (μL/min/mg prot)	8.2
	Cl_{int} HH (μL/min/10^6 cells)	4.5
	Cl_{int} MH (μL/min/10^6 cells)	41.3

Supplementary Table S3. Genetically modified cell lines for specificity tests

Receptor	Accession Number	Assay	Cell line	Reference agonist
**human FFA1 (GPR40)**	AAI20945.1	Aequorin	CHO-K1-mt aequorin	DHA
**human FFA2 (GPR43)**	NP_005297.1	Aequorin	CHO-K1-mt aequorin	Propionate
**human FFA3 (GPR41)**	NP_005295.1	Aequorin	CHO-K1-mt aequorin	Propionate
**human FFA4 (GPR120)**	NP_001182684.1	Aequorin	CHO-K1-mt aequorin-G 16	Linolenic acid
**human HCA3 (GPR109B)**	ABX64359.1	Aequorin	CHO-K1-mt aequorin-G 16	L-lactate
**mouse GPR84**	NP_109645.1	cAMP	CHO-K1-mt aequorin-Gqi5	Capric Acid

Supplementary Table S4. Specificity Tests – ZQ-16

Test	Concentration (nM)	% Activation 1	% Activation 2	% Activation Average
One concentration test, agonist mode GPR40 (FFA1) receptor, Aequorin assay	10000	1.40	-0.26	0.57
One concentration test, agonist mode GPR43 (FFA2) receptor, Aequorin assay	10000	0.15	-0.11	0.02
One concentration test, agonist mode GPR41 receptor (FFA3), Aequorin assay	10000	0.59	-0.17	0.21
One concentration test, agonist mode GPR120 (FFA4) receptor, Aequorin assay	10000	-1.67	-1.78	-1.72
One concentration test, agonist mode HCA3 receptor, Aequorin assay	10000	0.64	-0.30	0.17
**Test**	**% Activation Average at maximal concentration**	**EC50 (nM)**	**Hill Coefficient**	**Top (%)**
Dose-response test, Agonist mode mouse GPR84 receptor, cAMP assay	100.49	2.02	0.97	103.52

Supplementary Table S5. Results SAFETYscan E/IC50 ELECT - ZQ-16

Target Class	Assay Name	Assay Target	Mode	Assay Target_Mode	ZQ-16 RC50 (uM)	ZQ-16 Max Reponse
GPCR	Calcium Flux	ADORA2A	Agonist	ADORA2A_Agonist	0.08702	102.87
GPCR	Calcium Flux	ADRA1A	Agonist	ADRA1A_Agonist	>30	0
GPCR	Calcium Flux	AVPR1A	Agonist	AVPR1A_Agonist	>30	2.61
GPCR	Calcium Flux	CCKAR	Agonist	CCKAR_Agonist	>30	0.22
GPCR	Calcium Flux	CHRM1	Agonist	CHRM1_Agonist	>30	1.3
GPCR	Calcium Flux	CHRM3	Agonist	CHRM3_Agonist	>30	0
GPCR	Calcium Flux	EDNRA	Agonist	EDNRA_Agonist	>30	0
GPCR	Calcium Flux	HRH1	Agonist	HRH1_Agonist	>30	1.94
GPCR	Calcium Flux	HTR2A	Agonist	HTR2A_Agonist	>30	1.08
GPCR	Calcium Flux	HTR2B	Agonist	HTR2B_Agonist	>30	0
GPCR	Calcium Flux	ADORA2A	Antagonist	ADORA2A_Antagonist	>30	40.52
GPCR	Calcium Flux	ADRA1A	Antagonist	ADRA1A_Antagonist	>30	10
GPCR	Calcium Flux	AVPR1A	Antagonist	AVPR1A_Antagonist	>30	0
GPCR	Calcium Flux	CCKAR	Antagonist	CCKAR_Antagonist	>30	3.85
GPCR	Calcium Flux	CHRM1	Antagonist	CHRM1_Antagonist	>30	1.6
GPCR	Calcium Flux	CHRM3	Antagonist	CHRM3_Antagonist	>30	7.73
GPCR	Calcium Flux	EDNRA	Antagonist	EDNRA_Antagonist	>30	0
GPCR	Calcium Flux	HRH1	Antagonist	HRH1_Antagonist	>30	6.32
GPCR	Calcium Flux	HTR2A	Antagonist	HTR2A_Antagonist	>30	0
GPCR	Calcium Flux	HTR2B	Antagonist	HTR2B_Antagonist	>30	6.9
GPCR	cAMP	ADRA2A	Agonist	ADRA2A_Agonist	>30	18.72
GPCR	cAMP	ADRB1	Agonist	ADRB1_Agonist	>30	0.23
GPCR	cAMP	ADRB2	Agonist	ADRB2_Agonist	>30	0.34

**Target Class**	**Assay Name**	**Assay Target**	**Mode**	**Assay Target_Mode**	**ZQ-16 RC50 (uM)**	**ZQ-16 Max Reponse**
GPCR	cAMP	CHRM2	Agonist	CHRM2_Agonist	>30	26.39
GPCR	cAMP	CNR1	Agonist	CNR1_Agonist	>30	8.35
GPCR	cAMP	CNR2	Agonist	CNR2_Agonist	11.47334	86.97
GPCR	cAMP	DRD1	Agonist	DRD1_Agonist	>30	0
GPCR	cAMP	DRD2S	Agonist	DRD2S_Agonist	>30	16.19
GPCR	cAMP	HRH2	Agonist	HRH2_Agonist	>30	0.33
GPCR	cAMP	HTR1A	Agonist	HTR1A_Agonist	>30	13.41
GPCR	cAMP	HTR1B	Agonist	HTR1B_Agonist	>30	15.07
GPCR	cAMP	OPRD1	Agonist	OPRD1_Agonist	>30	35.32
GPCR	cAMP	OPRK1	Agonist	OPRK1_Agonist	>30	9.49
GPCR	cAMP	OPRM1	Agonist	OPRM1_Agonist	>30	26.86
GPCR	cAMP	ADRA2A	Antagonist	ADRA2A_Antagonist	>30	0
GPCR	cAMP	ADRB1	Antagonist	ADRB1_Antagonist	>30	33.52
GPCR	cAMP	ADRB2	Antagonist	ADRB2_Antagonist	18.87295	55.39
GPCR	cAMP	CHRM2	Antagonist	CHRM2_Antagonist	>30	0
GPCR	cAMP	CNR1	Antagonist	CNR1_Antagonist	>30	0
GPCR	cAMP	CNR2	Antagonist	CNR2_Antagonist	>30	0
GPCR	cAMP	DRD1	Antagonist	DRD1_Antagonist	>30	22.76
GPCR	cAMP	DRD2S	Antagonist	DRD2S_Antagonist	>30	0
GPCR	cAMP	HRH2	Antagonist	HRH2_Antagonist	>30	26.52
GPCR	cAMP	HTR1A	Antagonist	HTR1A_Antagonist	>30	8.61
GPCR	cAMP	HTR1B	Antagonist	HTR1B_Antagonist	>30	0.4
GPCR	cAMP	OPRD1	Antagonist	OPRD1_Antagonist	>30	0
GPCR	cAMP	OPRK1	Antagonist	OPRK1_Antagonist	>30	0
GPCR	cAMP	OPRM1	Antagonist	OPRM1_Antagonist	>30	0
Ion Channel	Ion Channel	CAV1.2	Blocker	CAV1.2_Blocker	>30	0
Ion Channel	Ion Channel	GABAA	Blocker	GABAA_Blocker	>30	9.19
Ion Channel	Ion Channel	hERG	Blocker	hERG_Blocker	>30	0
Ion Channel	Ion Channel	HTR3A	Blocker	HTR3A_Blocker	>30	0
Ion Channel	Ion Channel	KvLQT1/minK	Blocker	KvLQT1/minK_Blocker	>30	24.44
Ion Channel	Ion Channel	nAChR(a4/b2)	Blocker	nAChR(a4/b2)_Blocker	>30	0
Ion Channel	Ion Channel	NAV1.5	Blocker	NAV1.5_Blocker	>30	0
Ion Channel	Ion Channel	NMDAR (1A/2B)	Blocker	NMDAR (1A/2B)_Blocker	>30	0
Ion Channel	Ion Channel	GABAA	Opener	GABAA_Opener	>30	3.11
Ion Channel	Ion Channel	HTR3A	Opener	HTR3A_Opener	>30	5.89
Ion Channel	Ion Channel	KvLQT1/minK	Opener	KvLQT1/minK_Opener	>30	0
Ion Channel	Ion Channel	nAChR(a4/b2)	Opener	nAChR(a4/b2)_Opener	>30	0
Ion Channel	Ion Channel	NMDAR (1A/2B)	Opener	NMDAR (1A/2B)_Opener	>30	6.01
Kinases	Binding	INSR	Inhibitor	INSR_Inhibitor	>30	12.19
Kinases	Binding	LCK	Inhibitor	LCK_Inhibitor	>30	0.76
Kinases	Binding	ROCK1	Inhibitor	ROCK1_Inhibitor	>30	15.07
Kinases	Binding	VEGFR2	Inhibitor	VEGFR2_Inhibitor	>30	16.24
NHR	NHR Nuclear Translocation	AR	Agonist	AR_Agonist	>30	0

Target Class	Assay Name	Assay Target	Mode	Assay Target_Mode	ZQ-16 RC50 (uM)	ZQ-16 Max Reponse
NHR	NHR Nuclear Translocation	AR	Antagonist	AR_Antagonist	>30	0.66
NHR	NHR Protein Interaction	GR	Agonist	GR_Agonist	>30	0.3
NHR	NHR Protein Interaction	GR	Antagonist	GR_Antagonist	>30	7.42
Non-Kinase Enzymes	Enzymatic	AChE	Inhibitor	AChE_Inhibitor	>30	0
Non-Kinase Enzymes	Enzymatic	COX1	Inhibitor	COX1_Inhibitor	>30	10.56
Non-Kinase Enzymes	Enzymatic	COX2	Inhibitor	COX2_Inhibitor	>30	6.5
Non-Kinase Enzymes	Enzymatic	MAOA	Inhibitor	MAOA_Inhibitor	>30	6.39
Non-Kinase Enzymes	Enzymatic	PDE3A	Inhibitor	PDE3A_Inhibitor	>30	0
Non-Kinase Enzymes	Enzymatic	PDE4D2	Inhibitor	PDE4D2_Inhibitor	>30	6.93
Transporter	Transporter	DAT	Blocker	DAT_Blocker	6.88557	63.37
Transporter	Transporter	NET	Blocker	NET_Blocker	>30	23.98
Transporter	Transporter	SERT	Blocker	SERT_Blocker	>30	6.07

Supplementary Table S6. Administration Scheme PK studies 6-OAU

Study Variables	Group 1	Group 2	Group 3
**Animal Species/ Strain/ Sex**	Mice/C57BL/6/ Female (20-35g)		
**(Body Weight)**			
**Test Item**	6-OAU		
**Number of Animals**	9	9	9
**Route of Administration**	IV	PO	IP
**Feeding Condition**	Fed	Overnight Fasting, feeding 4 h post dose	Overnight Fasting, feeding 4 h post dose
**Dose (mg/kg)**	1	10	3
**Dose Volume (mL/kg)**	2	10	5
**Concentration (mg/mL)**	0.5	1	0.6
**Formulation Vehicle**		DMSO (5% v/v) + Solutol (15%v/v) +23%w/v Captisol in Water (80% v/v)	
**Sample/Collection - Type**	Blood/saphenous vein		
**Anti-coagulant**	Li Heparin		
**Time points**	0.083, 0.25, 0.5, 1, 2, 4, 8 and 24 h	0.25, 0.5, 1, 2, 4, 6, 8 and 24 h	0.25, 0.5, 1, 2, 4, 6, 8 and 24 h

Supplementary Table S7. Administration Scheme PK studies ZQ-16

Study Variables	Group 1	Group 2	Group 3	Group 4
**Animal Species/ Strain/ Sex**	Mice/ C57BL/6/ Female (20-35g)			
**(Body Weight)**				
**Test Item**	ZQ-16			
**Number of Animals**	9	9	9	9
**Route of Administration**	IV	PO	IP	IP
**Feeding Condition**	Fed	Overnight Fasted: feeding 4 h post dose		
**Dose (mg/kg)**	1	10	3	0.457
**Dose Volume (mL/kg)**	5	10	10	10
**Concentration (mg/mL)**	0.2	1	0.3	0.0457

**Formulation Vehicle**	PEG400 (40%v/v) + PG (30%v/v) + WFI(30%v/v)		DMSO : Saline (2µl of 100mM stock+ 998µl of Saline)
**Sample/Collection - Type**	Blood/Terminal (Retro Orbital)		
**Anti-coagulant**	Li Heparin		
**Time points**	0.083, 0.25, 0.5, 1, 2, 4, 8 and 24h	0.25, 0.5, 1, 2, 4, 6, 8 and 24h	

Supplementary Table S8. Results PK study 6-OAU Plasma concentration (ng/mL)

**Vehicle**	**DMSO (5% v/v) + Solutol (15%v/v) +23%w/v Captisol in Water (80% v/v)**	**DMSO (5% v/v) + Solutol (15%v/v) +23%w/v Captisol in Water (80% v/v)**	**DMSO (5% v/v) + Solutol (15%v/v) +23%w/v Captisol in Water (80% v/v)**
**IV**	1 mpk		
**Cl (mL/min/kg)**<br>**V_{ss} (L/kg)**<br>**$T_{1/2}$ (h)**	50<br>1.2<br>0.4		
		**IP 3mpk**	**PO 10mpk**
**C_{max} (µM)**<br>**T_{max} (h)**<br>**AUC_{last} (µM*h)**<br>**F (%)**		0.5<br>1.0<br>0.7<br>17	2.3<br>1.0<br>3.0<br>21

Supplementary Table S9. Results PK study ZQ-16 Plasma concentration (ng/mL)

**Vehicle**	**PEG400 (40%)**<br>**PG (30%)**<br>**H_2O (30%)**	**DMSO/Saline**<br>**(2uL of 100mM**<br>**+ 998uL Saline)**	**PEG400 (40%)**<br>**PG (30%)**<br>**H_2O (30%)**	**PEG400 (40%)**<br>**PG (30%)**<br>**H_2O (30%)**
**IV**	1 mpk			
**Cl (mL/min/kg)**<br>**V_{ss} (L/kg)**<br>**$T_{1/2}$ (h)**	23<br>1.3<br>2.6			
		**IP 0.457 mpk**	**IP 3mpk**	**PO 10mpk**
**C_{max} (µM)**<br>**T_{max} (h)**<br>**AUC_{last} (µM*h)**<br>**F (%)**		14.3<br>0.3<br>3.9<br>277	4.4<br>0.3<br>5.6<br>62	26.6<br>0.3<br>13.6<br>47

Supplementary Figures

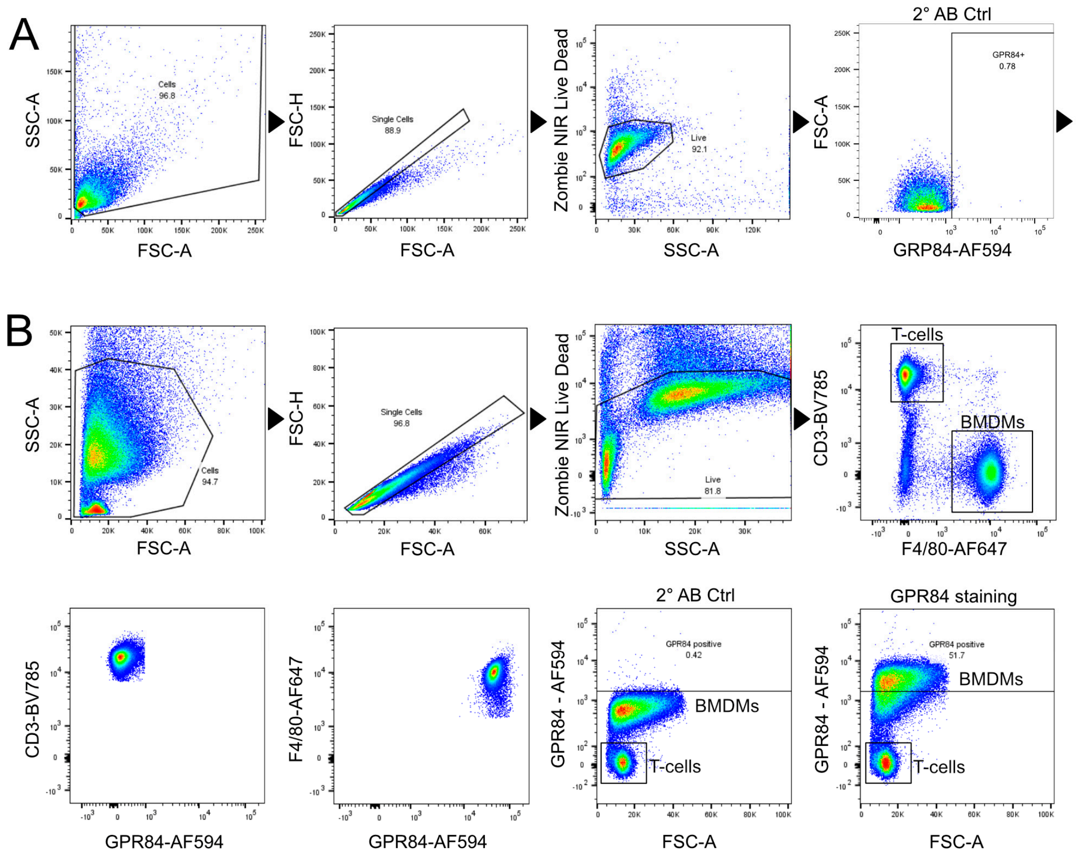

Supplementary Figure S1. Gating GPR84 staining. A) GRP84 surface expression on THP-1 cells and BMDMs was analyzed using flow cytometry. Manual gating on live single cells was performed using a secondary antibody control (2°AB Ctrl). B) Specificity of the anti-GRP84 antibody was tested mixing T cells isolated form mouse spleen and BMDMs. Manual gating on live single cells was performed using a secondary antibody control (2°AB Ctrl). Since the GPR84 signal was only detectable on BMDMs, which are known to express GPR84 on their surface but not on T-cells, which are lacking GPR84 surface expression, the antibody was considered specific for *in vitro* tests.

Compound 6-OAU: UPLC-MS (Reaction Neut 20% ESI_1): Rt = 2.52 min (99.0%); m/z 240.2 [M+H]+, m/z 238.0 [M-H]-.

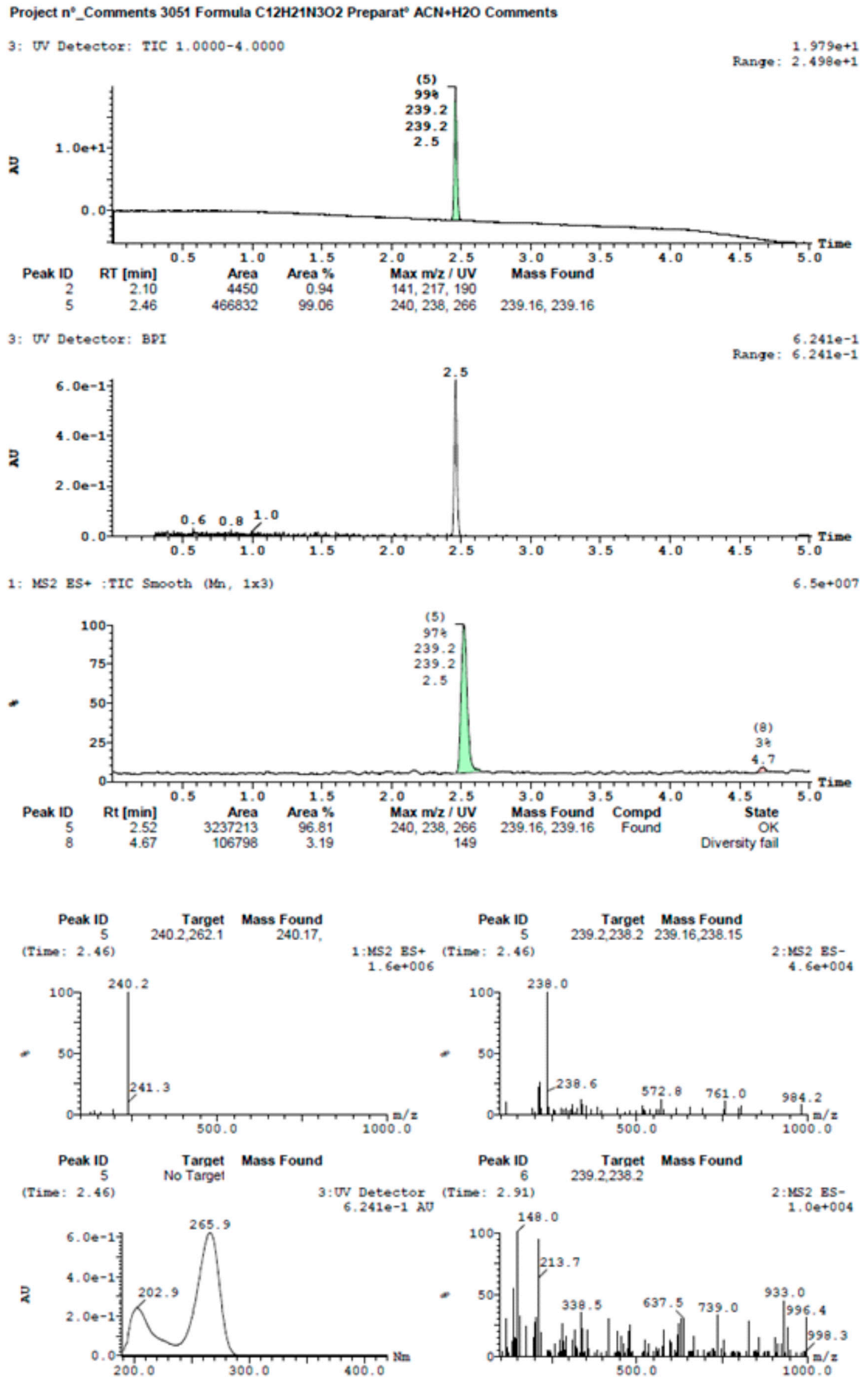

Supplementary Figure S2. 6-OAU Quality Control. The quality and purity of the synthetized 6-OAU was tested using mass spectrometry. Quality control was performed for every batch.

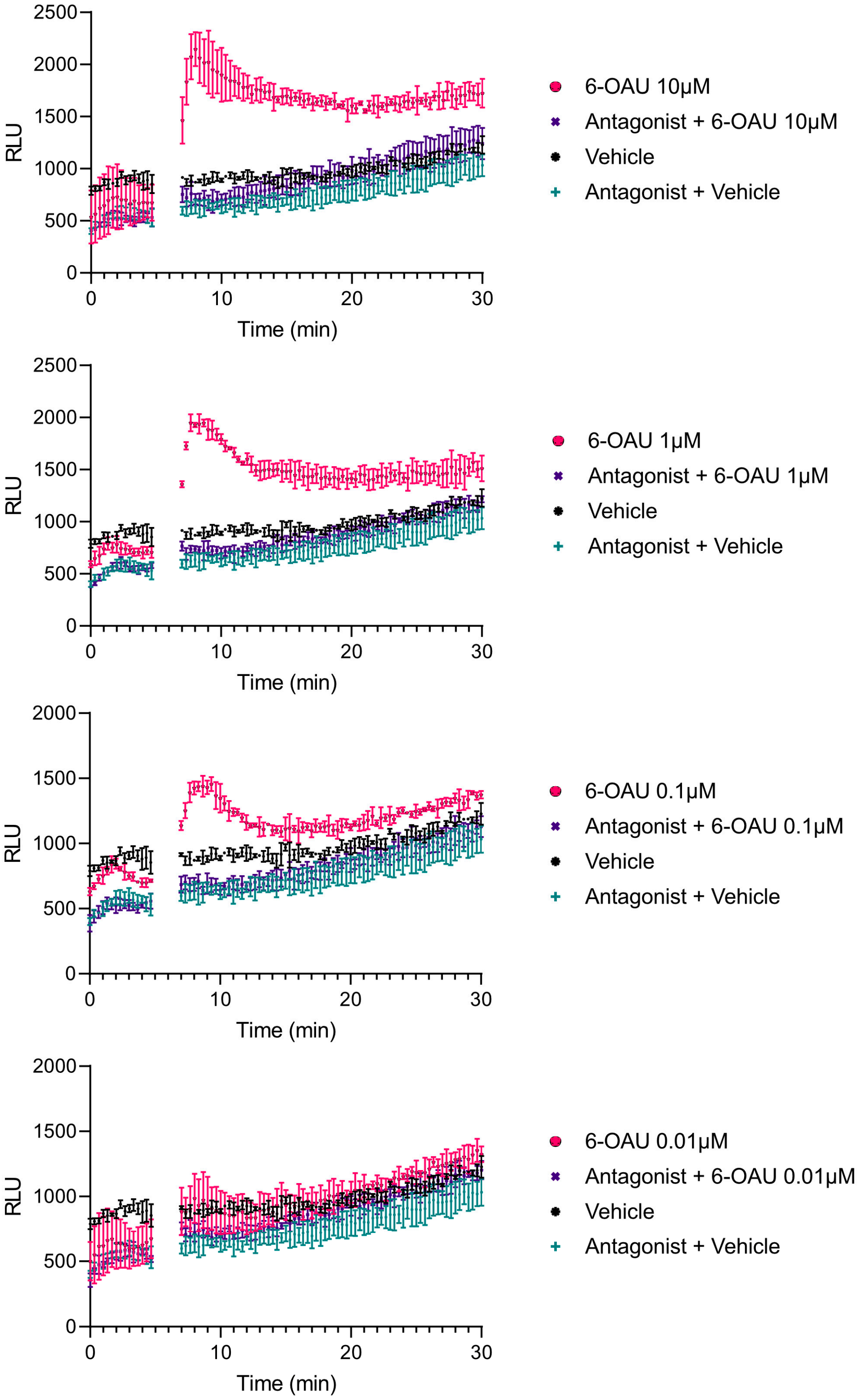

Supplementary Figure S3. 6-OAU induced ROS production in THP-1 cells. Differentiated THP-1 cells were pre-incubated with the GPR84 antagonist GLPG1205 or DMSO before stimulation with ZQ-16 at 0.01, 0.1, 1 and 10 μM. Controls were treated with DMSO instead. ROS production was quantified over time in an isoluminol-based assay. One representative experiment out of three is shown.

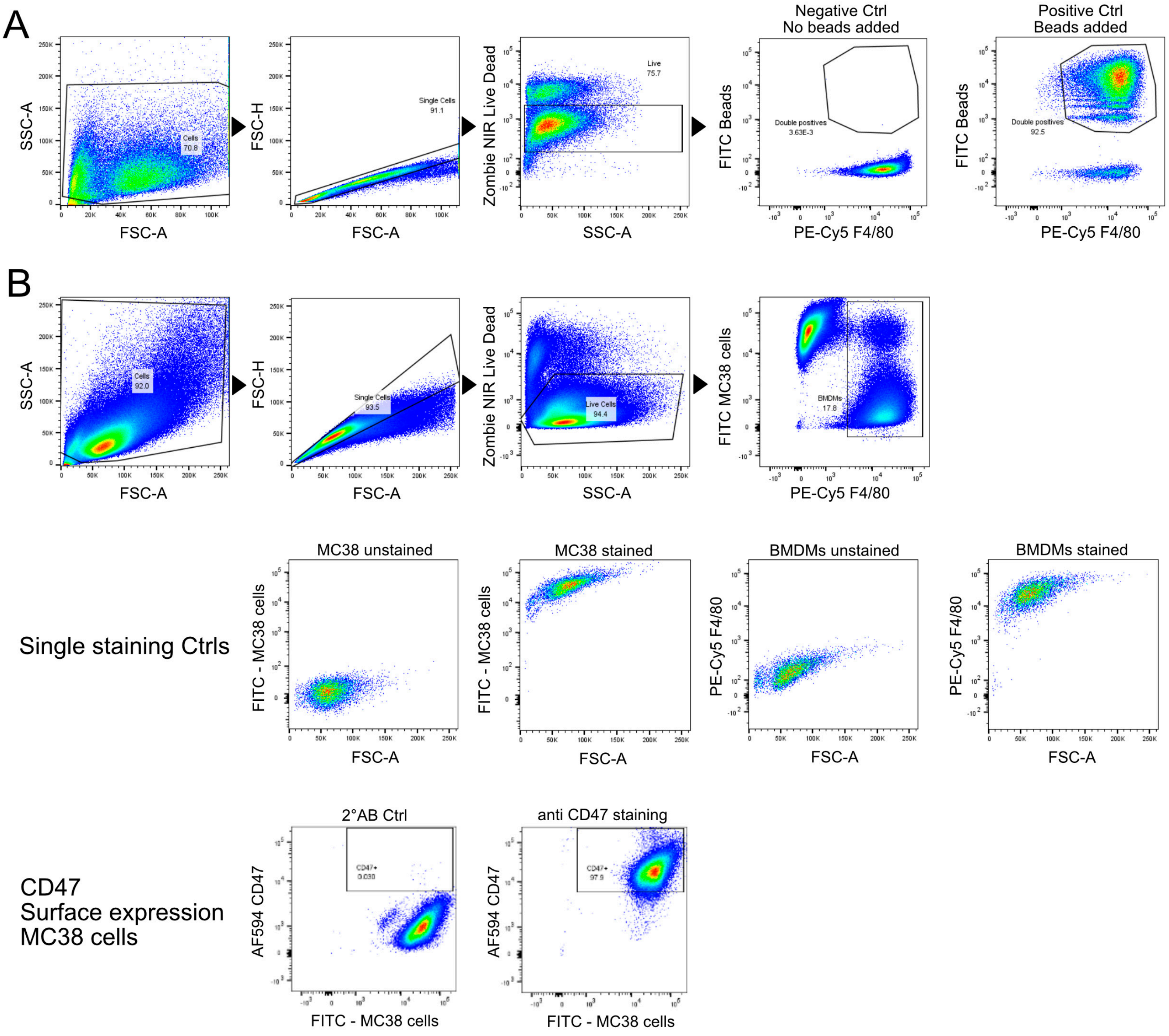

Supplementary Figure S4. Gating used in phagocytosis assays. A) Bead phagocytosis was quantified using flow cytometry. Manual gating on live single cells was performed using a negative control were no beads were added. B) MC38 ADCP was quantified using flow cytometry. Manual gating on live single cells was performed using single stained controls. The surface expression of CD47 was determined using a secondary antibody-stained control (2°AB Ctrl).

Compound ZQ-16: UPLC-MS (Reaction Neut 20% ESI_1): Rt = 2.48 min (100.0%); m/z 229.1 [M+H]+, m/z 227.2 [M-H]-.

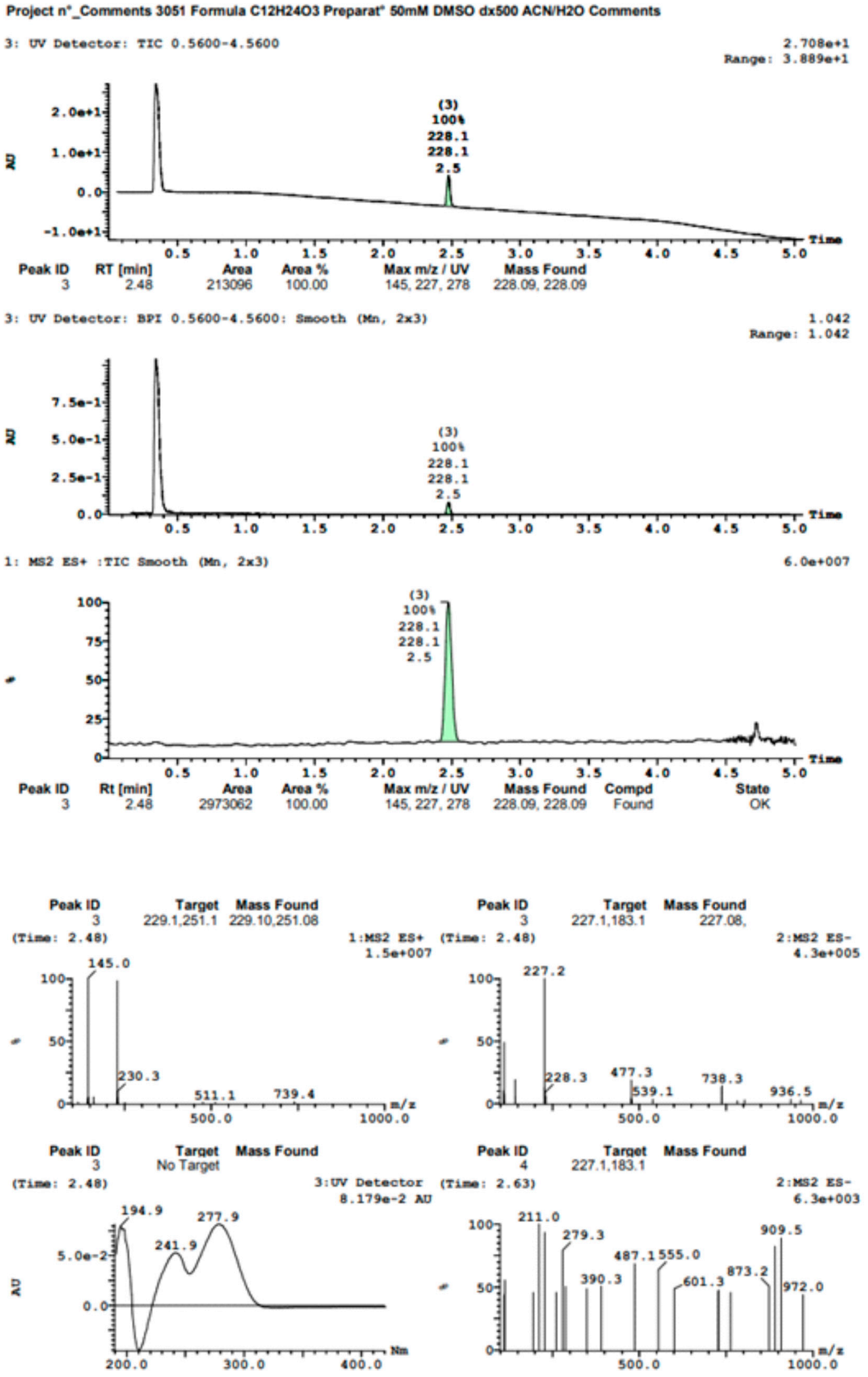

Supplementary Figure S5. ZQ-16 Quality Control. The quality and purity of the synthetized ZQ-16 was tested using mass spectrometry. Quality control was performed for every batch.

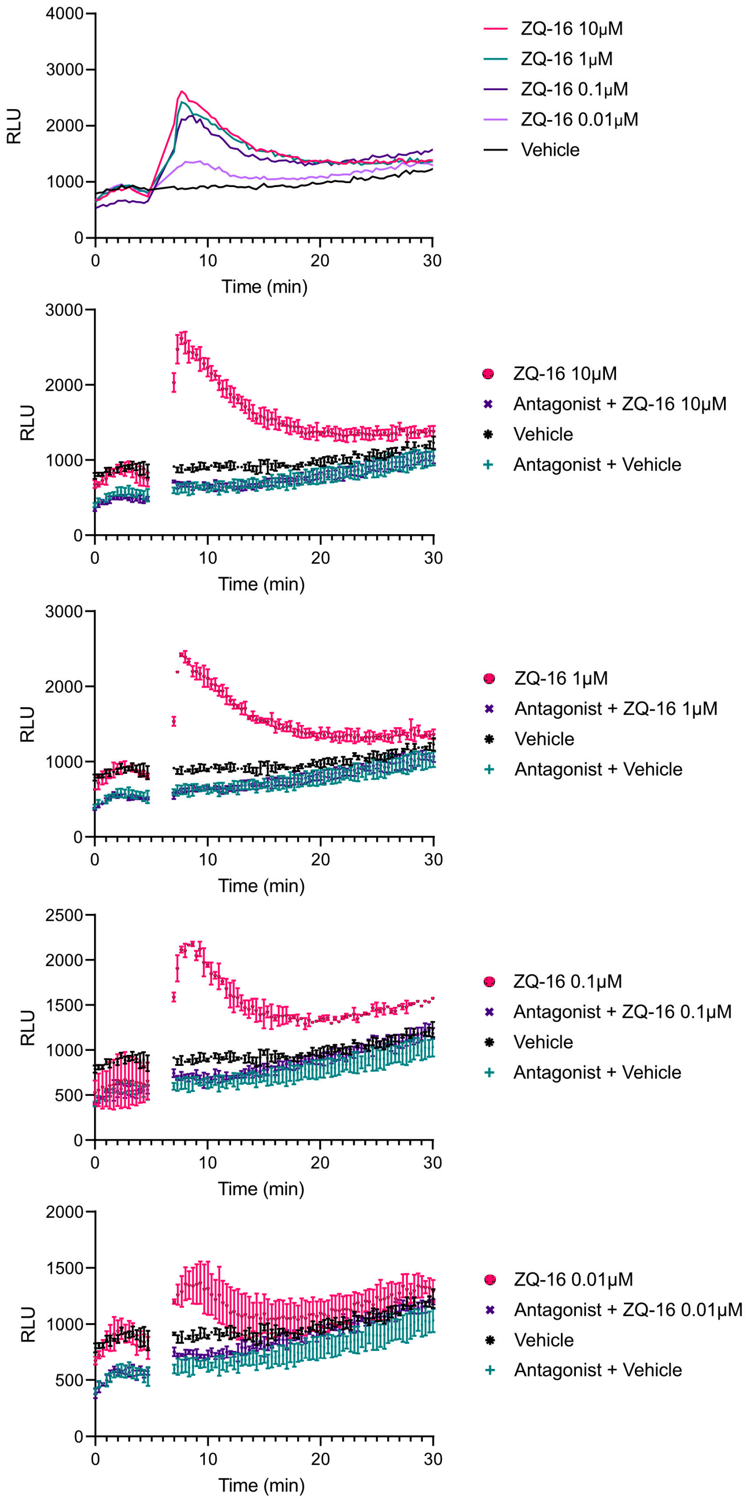

Supplementary Figure S6. ZQ-16 induced ROS production in THP-1 cells. Differentiated THP-1 cells were pre-incubated with the GPR84 antagonist GLPG1205 or DMSO before stimulation with ZQ-16 at 0.01, 0.1, 1 and 10 μM. Controls were treated with DMSO instead. ROS production was quantified over time in an isoluminol-based assay. One representative experiment out of three is shown.

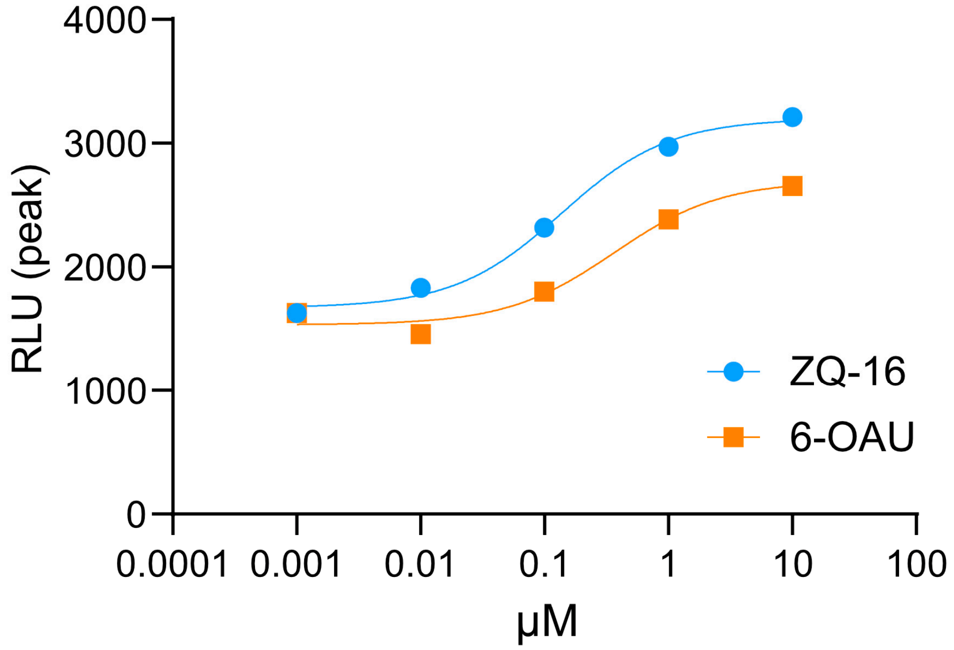

	ZQ-16	6-OAU
[Agonist] vs. response (three parameters)		
Best-fit values		
Bottom	1670	1533
Top	3199	2696
EC50 µM	0.1377	0.3640

Supplementary Figure S7. ZQ-16 was a more potent GPR84 agonist than 6-OAU. Dose-response curves and EC50 comparing the potency of ZQ-16 and 6-OAU on ROS production in THP-1 macrophages.

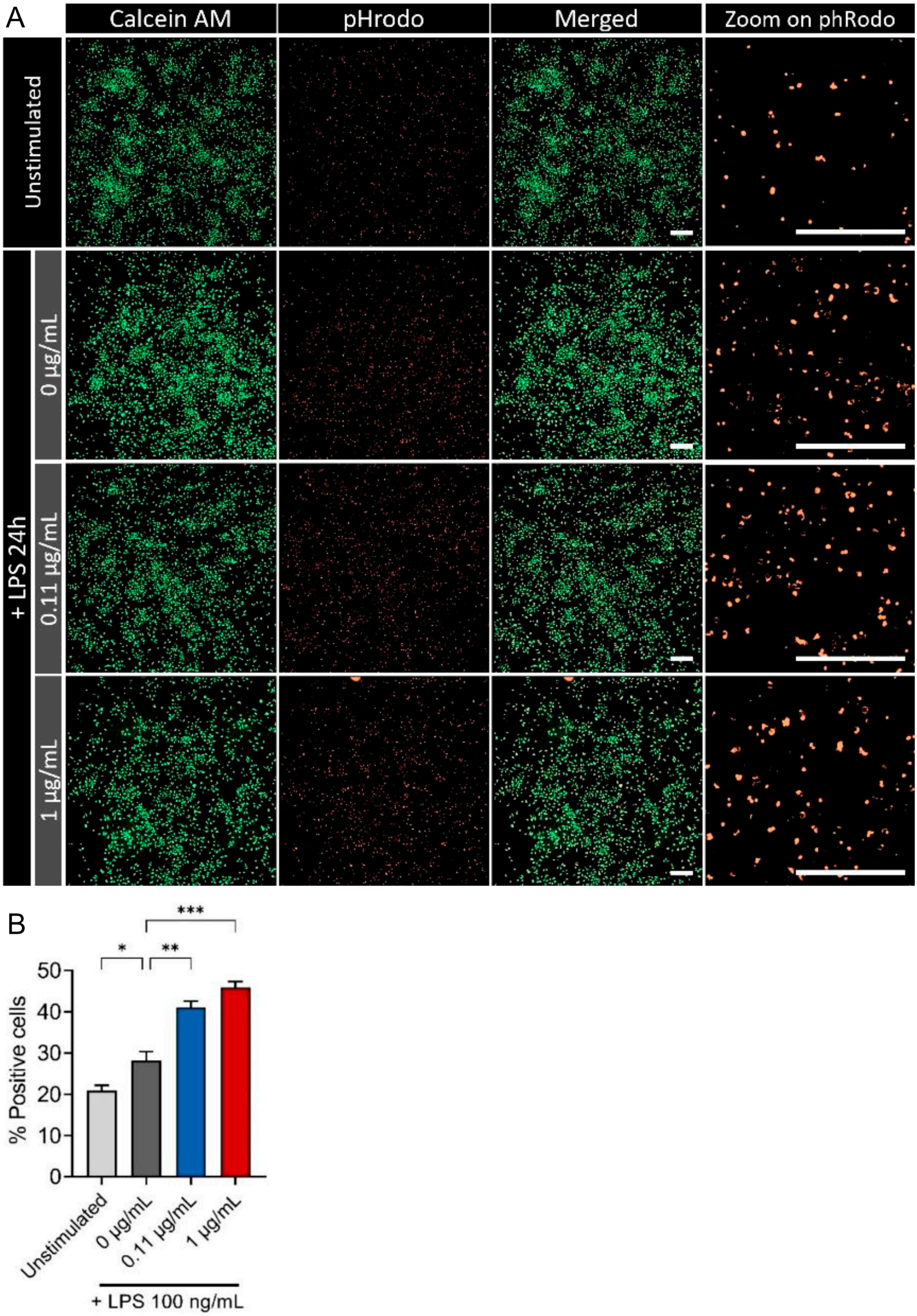
A
Calcein AM
pHrodo
Merged
Zoom on phRodo
Unstimulated
+ LPS 24h
0 µg/mL
0.11 µg/mL
1 µg/mL
B
***
*
**
% Positive cells
50
40
30
20
10
0
Unstimulated
0 µg/mL
0.11 µg/mL
1 µg/mL
+ LPS 100 ng/mL

Supplementary Figure S8. ADCP controls. (A) Representative images of the effect of LPS stimulation (100 ng/mL) for 24 h in the absence or presence of Magrolimab (anti-CD47) at two different concentrations (total: 0, 0.11 and 1 μg/mL) on J774-mediated phagocytosis of target Raji cells. Effector J774 macrophages were stained with calcein AM and Raji target cells were stained with Incucyte® pHrodo for 1 h prior to co-culture. Images were captured at 6 h after J774:Raji co-culture and analysis performed in the ImageXpress® Pico. Scale bar: 300 μm. (B) Graphs represent the percentage of Raji cells positive for phagocytosis normalized to the respective total number of macrophages. Each data point represents the average of three technical repeats (n=3 ± SEM). Statistical significance was determined by one-way ANOVA, followed by Dunnett's multiple comparisons post-hoc test: *** $p \leq 0.001$, ** $p \leq 0.01$, *$p < 0.05$.

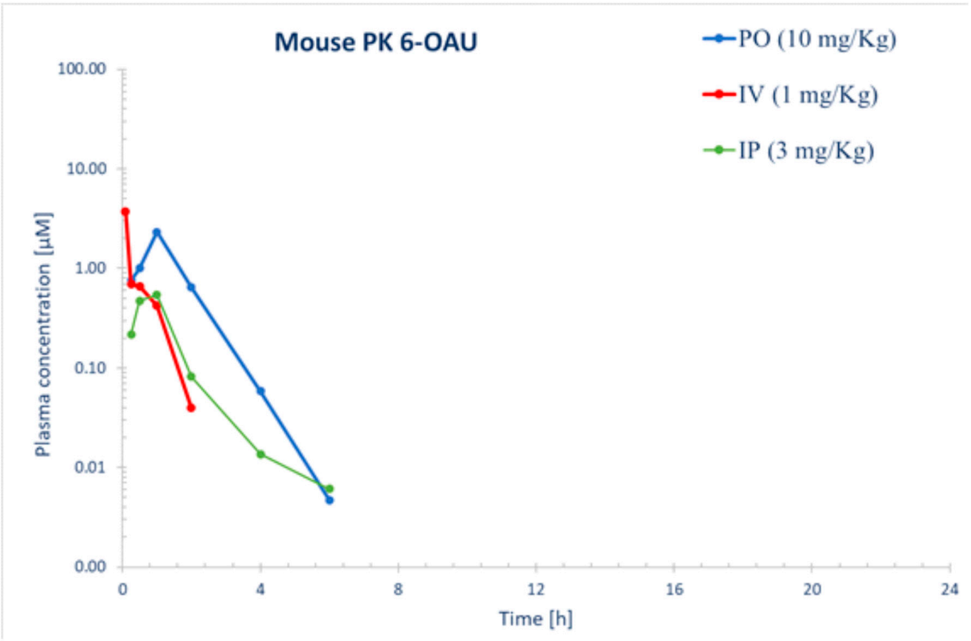

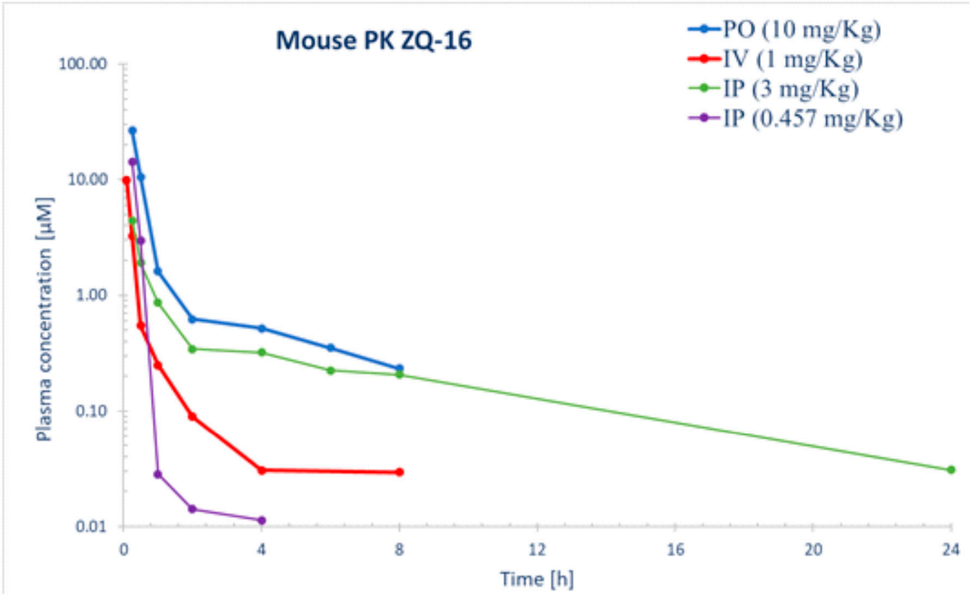

Supplementary Figure S9. PK studies. The pharmacokinetics of 6-OAU and ZQ-16 were assessed in vivo. GPR84 agonists were administered per oral gavage (PO), or per intravenous (IV) and intraperitoneal (IP) injections. And plasma concentrations of 6-OAU and ZQ-16 were determined over time.
